# Synthesis and Biological Evaluation of Coumarin-Linked 4-Anilinomethyl-1,2,3-Triazoles as Potent Inhibitors of Carbonic Anhydrases IX and XIII Involved in Tumorigenesis

**DOI:** 10.3390/metabo11040225

**Published:** 2021-04-07

**Authors:** Pavitra S. Thacker, Prerna L. Tiwari, Andrea Angeli, Danaboina Srikanth, Baijayantimala Swain, Mohammed Arifuddin, Claudiu T. Supuran

**Affiliations:** 1 Department of Medicinal Chemistry, National Institute of Pharmaceutical Education and Research (NIPER), Balanagar, Hyderabad 500037, Telangana, India; thackerp42@yahoo.com (P.S.T.); tiwariprerna1997@gmail.com (P.L.T.); srikanthdanaboina1997@gmail.com (D.S.); baijayantimala87@gmail.com (B.S.); 2 Neurofarba Department, Sezione di Scienze Farmaceutiche e Nutraceutiche, Università degli Studi di Firenze, Via Ugo Schiff 6, 50019 Sesto Fiorentino, Florence, Italy; andrea.angeli@unifi.it; 3 Department of Chemistry, Anwarul Uloom College, 11-3-918, New Malleypally, Hyderabad 500001, Telangana, India

**Keywords:** carbonic anhydrases, coumarin, hCA IX, hCA XIII, 1,2,3-triazole

## Abstract

A series of coumarin-linked 4-anilinomethyl-1,2,3-triazoles (**6a**–**t**) was synthesized via a molecular hybridization approach, through carbon C-6 of the coumarin moiety. The synthesized compounds were evaluated for their inhibition of carbonic anhydrase (CA) isoforms I, II, IX and XIII. CAs IX and XIII were selectively inhibited over the off-target isoforms I and II. The best inhibitory profiles against CA IX were shown by compounds **6a**, **6e** and **6f** (K_i_ < 50 nM), with compound **6e** displaying the best inhibition with a K_i_ value of 36.3 nM. Compounds **6a**, **6b**, **6j**, **6o** and **6q** exhibited the best inhibitory profiles against CA XIII (K_i_ < 100 nM). These compounds can be further explored for the discovery of potent and effective CA IX and CA XIII inhibitors.

## 1. Introduction

Metalloenzymes and metalloproteins are two key players in cellular regulation, which act by maintenance of homoeostasis, control of metal ion concentrations, buffering and activation of signal pathways. Carbonic anhydrases (CAs, EC 4.2.1.1) are a super family of metalloenzymes, which play a fundamental role in the inter conversion of CO_2_ to bicarbonate and proton. Thereby, they perform the constant physiological demand of converting CO_2_ generated during metabolic oxidative processes into ionic, soluble species [1,2].

These enzymes have evolved convergently over the eons into eight families, which are genetically distinct. The families are designated as α, β, γ, δ, ζ, η, θ and ι CAs. All CAs exhibit catalytic activity, on account of a metal ion present within their active sites. Most of them contain a Zn(II) ion in their active sites, but in ζ CAs the Zn(II) ion is interchangeable with Cd(II). Similarly, Co(II) may substitute Zn(II) in α CAs and Fe(II) might be present in γ CAs, especially under anaerobic conditions. The human CAs belong to α class (denoted as human carbonic anhydrases (hCAs)), consisting of sixteen isozymes differing in various aspects like structure, cellular localization, organ and tissue distribution, catalytic properties and response to various inhibitors. The hCAs I, II, III, VII, VIII, X and XIII are cytosolic, hCAs IV, IX, XII, XIV and XV are membrane-bound, hCAs VA and VB are mitochondrial and hCA VI is secretory in nature [3,4].

Hypoxia has been determined to be one of the key features of several solid tumors, due to incomplete vascularization and overexpression of proteins which dramatically change the metabolism. Hypoxia promotes tumor-adaptive processes such as acidosis, tumor aggressiveness, tumor progression and resistance to treatment. The key player in such processes is a transcription factor, Hypoxia-Inducible Factor (HIF), in particular isoform HIF-1 (but also HIF-2). It induces several genetic as well as epigenetic changes, along with controlling major aspects of cell proliferation and metastasis. It also plays an important role in regulation of metabolism and acidosis induced due to hypoxia [5,6,7,8].

Among the various hCA isoforms mentioned above, the transmembrane isoform, hCA IX, is over-expressed in tumor cells (particularly hypoxic tumors) while being restrictively expressed in normal cells. This over-expression is the result of the transcriptional activation by the HIF-1 pathway, mediated by the hypoxic microenvironment of the tumor cells. The significant role of hCA IX has been established in promoting tumorigenesis, cancer cell signaling, invasiveness, metastasis, treatment resistance and acidification by modulation of pH caused due to tumor metabolism. The effective inhibition of this isoform can impair the growth of primary tumors as well as metastasis and also reduce the population of cancer stem cells [9,10,11,12,13].

On the other hand, hCA XIII is a cytosolic isoform, closely related to hCAs I, II and III. Compared to other organs, it is abundantly present in human reproductive organs, where it is thought to play a role in fertilization processes [14]. Recently, it has been shown to be involved in tumorigenesis too, but these aspects were poorly investigated to date [15].

Coumarins are primarily natural products that are widespread as secondary metabolites in plants, microorganisms, and marine organisms. They are bicyclic, aromatic compounds which have been known to possess diverse biological activities like anticoagulant, anticancer, antimicrobial, neuroprotective, anti-inflammatory, antidiabetic, and anticonvulsant activities [16,17]. About a decade ago, coumarins were discovered to exhibit “non-classical” inhibition of CAs, acting via a “prodrug”—like inhibition mechanism [18]. Indeed, the coumarins are initially acting as CA substrates, their lactone ring being hydrolyzed by the enzyme to 2-hydroxy-cinnamic acids which bind in a very unusual part of the CA active site, at its entrance, occluding it [16,17]. After this finding, several groups, all of them collaborating with ours, have reported diverse coumarin-containing molecules, which act as potent and frequently isoform-selective inhibitors of human CAs, particularly hCA IX and XII [19]. Supuran and co-workers have reported several natural coumarins as potent inhibitors of hCA XIII [20]. The 1,2,3-triazole, a key member of the “azole” family, constitutes a very important pharmacophore and privileged scaffold in medicinal chemistry. This combination accounts for advantageous chemical properties and diverse biological profiles [21]. In addition to this, 1,2,3-triazoles have also been studied extensively as fragments in CA inhibitors, being used as linkers in various classes of compounds which effectively inhibited hCAs, including hCA IX and XII [22].

**Figure 1:**
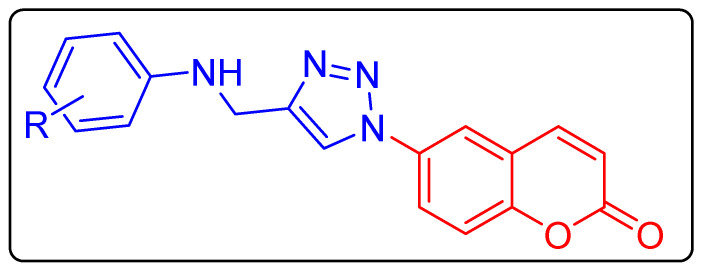
General structure of the compounds reported in the paper.

Hence, in continuation of our quest for finding selective and potent inhibitors of various CA isoforms, we synthesized coumarin-linked 4-anilinomethyl-1,2,3-triazoles, through carbon C-6 of the coumarin moiety (Figure 1). The synthesized compounds were evaluated against a panel of three cytosolic isoforms, hCAs I, II and XIII as well as against the transmembrane tumor-associated isoform, hCA IX, using Acetazolamide (AAZ) as the standard drug.

## 2. Results

### 2.1. Chemistry

**Scheme 1:**
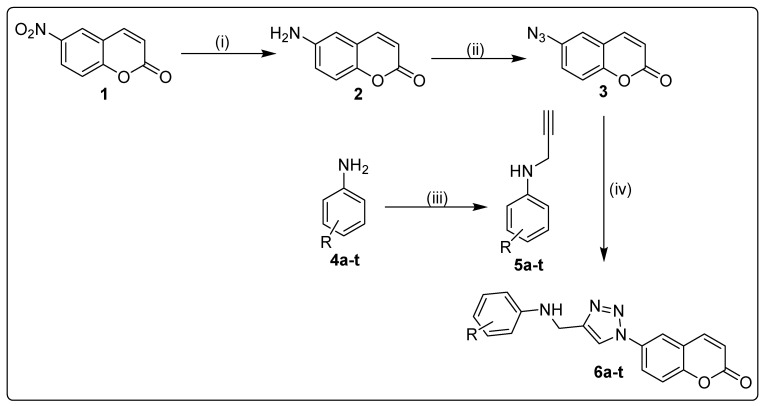
General synthetic scheme for the synthesis of coumarin-linked 4-anilinomethyl-1,2,3-triazoles (**6a**–**t**). Reagents and conditions: (i) Fe powder, NH_4_Cl, EtOH:H_2_O::3:1, 80 °C, 4–5 h (ii) (i) Conc. H_2_SO_4_, H_2_O, 0 °C (ii) NaNO_2_, 0 °C, 15 min (iii) NaN_3_, 0 °C-rt, 1–2 h (iii) Propargyl bromide, K_2_CO_3_, DMF, rt, 6 h (iv) CuSO_4_.5H_2_O, Sod. Ascorbate, t-BuOH:H_2_O::1:1, rt, 12 h, Yield 51–82%.

The synthesis of the coumarin-linked 4-anilinomethyl-1,2,3-triazoles (**6a**–**t**) was performed according to the general synthetic scheme as illustrated in Scheme 1. The reduction of 6-nitrocoumarin to 6-aminocoumarin (**2**) and the conversion of 6-aminocoumarin to 6-azidocoumarin (**3**) was carried out according to the reported methods [23,24]. On the other hand, various substituted anilines (**4a**–**t**) were subjected to propargylation using propargyl bromide, to yield N-propargylated anilines (**5a**–**t**) as per reported literature [25]. Finally, these N-propargylated anilines (**5a**–**t**) were reacted with 6-azidocoumarin (**3**) to afford the final compounds **6a**–**t**.

### 2.2. Carbonic Anhydrase Inhibition

The coumarin-linked 4-anilinomethyl-1,2,3-triazoles, **6a**–**t**, were tested for their inhibition of three cytosolic isoforms, namely hCAs I, II and XIII, as well as the transmembrane tumor-associated isoform, hCA IX. Acetazolamide (AAZ) was used as the standard drug. The following conclusions can be drawn from the results shown in Table 1:

- The newly synthesized compounds, **6a**–**t** were found to be ineffective against the cytosolic isoforms hCAs I and II (K_i_ > 50,000 nM).
- The transmembrane tumor-associated isoform hCA IX was inhibited by the compounds, **6a**–**t** in a low to moderate nanomolar range with the K_i_ values ranging from 36.3 to 642.8 nM. Compound **6e** possessing a 4-bromo substitution on the aniline moiety exhibited the most potent inhibition of hCA IX with a K_i_ value of 36.3 nM. Compound **6f** is possessing a 4-isopropyl substitution on the aniline moiety and compound **6a** possessing a 4-methyl substitution on the aniline moiety exhibited K_i_ values of 45.0 and 48.4 nM, respectively. Barring compounds **6p**, **6r** and **6t**, all the compounds displayed K_i_ values < 100 nM against hCA IX. Additionally, the compounds containing electron-donating substituents on the phenyl ring of aniline displayed better inhibitory profiles over the electron-withdrawing substituents.
- The cytosolic isoform hCA XIII was inhibited by the compounds **6a**–**t** in a low to high nanomolar range with the K_i_ values ranging from 90.1 to 8149 nM. Compound **6b** possessing an unsubstituted phenyl ring showed the highest inhibition of hCA XIII with a K_i_ value of 90.1 nM. The other compounds which exhibited K_i_ values < 100 nM are compounds **6j**, **6a**, **6o** and **6q** with K_i_ values of **91.6**, **92.6**, **95.4** and **96.7** nM, respectively.
- Thus, it is implied from the overall results that the compounds **6a**–**t** are highly selective inhibitors of hCAs IX and XIII over the off-target isoforms, hCAs I and II.

## 3. Experimental Section

### 3.1. General

All the chemicals and solvents were procured and utilized as such from the suppliers. Wherever necessary, anhydrous solvents were used. Thin Layer Chromatography (TLC) analysis was done by utilizing Merck silica gel 60 F_254_ aluminum plates. Stuart Digital Melting Point Apparatus (SMP 30) was used in determining the melting points of the compounds, which are uncorrected. ^1^H and ^13^C NMR spectra were recorded using Bruker Avance 500 MHz and 125 MHz, respectively, using DMSO-d_6_ as the solvent. Chemical Shift values are recorded in ppm using TMS as the internal standard. HRMS were determined by Agilent QTOF mass spectrometer 6540 series instrument and were performed using ESI techniques at 70 eV.

#### 3.1.1. Synthesis of 6-Amino-2H-Chromen-2-One (**2**)

To a reaction mixture of Fe powder (1.7 g, 31 mmol) and NH_4_Cl (0.8 g, 15 mmol) in EtOH (30 mL) and water (10 mL) was added 6- nitro-2H-chromen-2-one (1.5 g, 7.8 mmol). The reaction mixture was stirred at 80 °C for 4–5 h. Upon completion of the reaction as monitored by TLC, the reaction mixture was cooled to room temperature and it was extracted with ethyl acetate (3 × 100 mL). The organic layer was washed with brine and dried over Na_2_SO_4_. The solid was filtered off and the filtrate was concentrated under reduced pressure. The resulting crude product was purified by column chromatography using silica gel (60–120 mesh) as the stationary phase and EtOAc in hexane (0 percent to 20 percent) as the mobile phase to afford the entitled compound **2** as a yellow-colored solid (Yield: 85%).

#### 3.1.2. Synthesis of 6-Azido-2H-Chromen-2-One (**3**)

To a mixture of 2.5 mL of conc. H_2_SO_4_ and 7 mL of water was dissolved 1.6 g (10 mmol) of intermediate **2** and the solution was cooled to 0 °C. To this solution, a saturated solution of 0.83 g (12 mmol) of NaNO_2_ was added maintaining the temperature < 5 °C. After stirring for 5 min, a solution of 0.65 g (10 mmol) of NaN_3_ in 5 mL of water was added. The reaction mixture was allowed to stir at room temperature and it was monitored by TLC. Upon completion of the reaction as monitored by TLC, the solid obtained was filtered, dried and weighed to afford the entitled compound **3** as a cream-colored solid (Yield: 90%).

#### 3.1.3. Synthesis of N-Propargylated Anilines (**5a**–**t**)

To a solution of substituted anilines **4a**–**t** (4.0 equivalent) in 5–10 mL DMF, potassium carbonate (2.0 equivalent) was added. The mixture was stirred for 5 min at room temperature. Thereafter, a solution of propargyl bromide (1.0 equivalent) was added to the mixture drop-wise. The resulting mixture was stirred at room temperature for 6 h. After completion of reaction as indicated by TLC, crushed ice was added to the reaction mixture and the solid obtained was filtered. The resulting crude solid was purified by column chromatography using silica gel (60–120 mesh) as the stationary phase and EtOAc in hexane (0 percent to 20 percent) as the mobile phase to afford the title compounds **5a**–**t**.

#### 3.1.4. Synthesis of Coumarin-Triazole Hybrids (**6a**–**t**)

To a mixture of 6-azidocoumarin (1 equivalent) and intermediates **5a**–**t** (1 equivalent) in 6 mL of water/tert-butanol (1:1), was added sodium ascorbate (0.3 equivalent), followed by copper (II) sulfate pentahydrate (0.03 equivalent). The heterogeneous mixture was stirred overnight at room temperature, at which point it cleared and TLC analysis indicated complete consumption of the reactants. To the reaction mixture was added crushed ice and the solid obtained was collected by filtration. Thereafter, the solid was washed with cold water and dried in oven. It was purified by silica gel chromatography using silica gel (60–120 mesh) as the stationary phase and EtOAc:Hexane:1:1 as mobile phase to afford the final compounds **6a**–**t**.

**6-(4-((p-tolylamino)methyl)-1H-1,2,3-triazol-1-yl)-2H-chromen-2-one**(**6a**) Yield: 55%; Yellow solid; m.p: 150–152 °C; ^1^H NMR (500 MHz, DMSO-*d*_6_) δ 8.68 (s, 1H), 8.31 (d, *J* = 2.3 Hz, 1H), 8.15 (d, *J* = 9.6 Hz, 1H), 8.11 (dd, *J* = 8.9, 2.5 Hz, 1H), 7.62 (d, *J* = 8.9 Hz, 1H), 6.91 (d, *J* = 8.1 Hz, 2H), 6.63 (d, *J* = 9.6 Hz, 1H), 6.61 (d, *J* = 7.2 Hz, 2H), 5.91 (t, *J* = 5.7 Hz, 1H), 4.37 (d, *J* = 5.8 Hz, 2H), 2.15 (s, 3H); ^13^C NMR (125 MHz, DMSO-*d*_6_) δ 159.9, 153.3, 147.8, 146.5, 144.0, 133.4, 129.7, 125.0, 123.8, 121.6, 119.9, 119.9, 118.3, 118.1, 113.0, 39.3, 20.5; HRMS (ESI): *m/z* calcd for [M+H]^+^ C_19_H_17_N_4_O_2_ 333.1352; found 333.1444.

**6-(4-((phenylamino)methyl)-1H-1,2,3-triazol-1-yl)-2H-chromen-2-one**(**6b**) Yield: 51%; Yellow solid; m.p: 159–161 °C; ^1^H NMR (500 MHz, DMSO-*d*_6_) δ 8.71 (s, 1H), 8.32 (d, *J* = 2.5 Hz, 1H), 8.15 (d, *J* = 9.7 Hz, 1H), 8.11 (dd, *J* = 8.9, 2.5 Hz, 1H), 7.62 (d, *J* = 8.9 Hz, 1H), 7.09 (t, *J* = 7.8 Hz, 2H), 6.69 (d, *J* = 7.9 Hz, 2H), 6.63 (d, *J* = 9.6 Hz, 1H), 6.57 (t, *J* = 7.2 Hz, 1H), 6.14 (t, *J* = 5.7 Hz, 1H), 4.40 (d, *J* = 5.8 Hz, 2H); ^13^C NMR (125 MHz, DMSO-*d*_6_) δ 159.9, 153.3, 148.8, 147.6, 144.0, 133.3, 129.3, 123.8, 121.6, 120.0, 119.9, 118.3, 118.1, 116.6, 112.8, 39.0; HRMS (ESI): *m/z* calcd for [M+H]^+^ C_18_H_15_N_4_O_2_ 319.1195; found 319.1201.

**6-(4-(((4-methoxyphenyl)amino)methyl)-1H-1,2,3-triazol-1-yl)-2H-chromen-2-one**(**6c**) Yield: 67%; Yellow solid; m.p: 140–142 °C; ^1^H NMR (500 MHz, DMSO-*d*_6_) δ 8.69 (s, 1H), 8.32 (d, *J* = 2.6 Hz, 1H), 8.15 (d, *J* = 9.6 Hz, 1H), 8.11 (dd, *J* = 8.9, 2.6 Hz, 1H), 7.62 (d, *J* = 8.9 Hz, 1H), 6.73 (d, *J* = 8.9 Hz, 2H), 6.64 (dd, *J* = 9.2, 4.9 Hz, 3H), 5.72 (t, *J* = 5.6 Hz, 1H), 4.35 (d, *J* = 5.6 Hz, 2H), 3.64 (s, 3H); ^13^C NMR (125 MHz, DMSO-*d*_6_) δ 159.9, 153.3, 151.5, 147.9, 144.0, 143.0, 133.4, 123.8, 121.6, 120.0, 119.9, 118.3, 118.1, 115.0, 113.9, 55.7; HRMS (ESI): *m/z* calcd for [M+H]^+^ C_19_H_17_N_4_O_3_ 349.1301; found 349.1328.

**6-(4-(((4-chlorophenyl)amino)methyl)-1H-1,2,3-triazol-1-yl)-2H-chromen-2-one**(**6d**) Yield: 74%; Yellow solid; m.p: 136–138 °C; ^1^H NMR (500 MHz, DMSO-*d_6_*) δ 8.72 (s, 1H), 8.32 (d, *J* = 2.5 Hz, 1H), 8.15 (d, *J* = 9.6 Hz, 1H), 8.11 (dd, *J* = 8.9, 2.6 Hz, 1H), 7.62 (d, *J* = 8.9 Hz, 1H), 7.11 (d, *J* = 8.8 Hz, 2H), 6.69 (d, *J* = 8.8 Hz, 2H), 6.64 (d, *J* = 9.6 Hz, 1H), 6.41 (t, *J* = 5.7 Hz, 1H), 4.39 (d, *J* = 5.8 Hz, 2H); ^13^C NMR (125 MHz, DMSO-*d_6_*) δ 160.0, 153.3, 147.6, 147.2, 144.0, 133.3, 129.0, 123.9, 121.7, 119.9, 119.9, 118.3, 118.1, 114.2, 38.9; HRMS (ESI): *m/z* calcd for [M+H]^+^ C_18_H_14_ClN_4_O_2_ 353.0805; found 353.0823.

**6-(4-(((4-bromophenyl)amino)methyl)-1H-1,2,3-triazol-1-yl)-2H-chromen-2-one**(**6e**) Yield: 70%; Yellow solid; m.p: 207–209 °C; ^1^H NMR (500 MHz, DMSO-*d*_6_) δ 8.72 (s, 1H), 8.32 (d, *J* = 2.4 Hz, 1H), 8.15 (d, *J* = 9.6 Hz, 1H), 8.11 (dd, *J* = 8.9, 2.5 Hz, 1H), 7.62 (d, *J* = 8.9 Hz, 1H), 7.22 (d, *J* = 8.8 Hz, 2H), 6.64 (dd, *J* = 9.2, 4.3 Hz, 3H), 6.44 (t, *J* = 5.6 Hz, 1H), 4.39 (d, *J* = 5.7 Hz, 2H); ^13^C NMR (125 MHz, DMSO-*d*_6_) δ 159.9, 153.3, 148.0, 147.2, 144.0, 133.3, 131.8, 123.8, 121.7, 119.9, 119.9, 118.3, 118.1, 114.7, 107.3, 38.9; HRMS (ESI): *m/z* calcd for [M+H]^+^ C_18_H_14_BrN_4_O_2_ 397.0300; found 397.1072.

**6-(4-(((4-isopropylphenyl)amino)methyl)-1H-1,2,3-triazol-1-yl)-2H-chromen-2-one**(**6f**) Yield: 80%; Yellow solid; m.p: 152–155 °C; ^1^H NMR (500 MHz, DMSO-*d_6_*) δ 8.71 (s, 1H), 8.33 (d, *J* = 2.5 Hz, 1H), 8.15 (d, *J* = 9.6 Hz, 1H), 8.12 (dd, *J* = 8.9, 2.6 Hz, 1H), 7.62 (d, *J* = 8.9 Hz, 1H), 6.97 (d, *J* = 8.4 Hz, 2H), 6.62 (dd, *J* = 11.1, 9.1 Hz, 2H), 5.97 (t, *J* = 5.8 Hz, 1H), 4.37 (d, *J* = 5.8 Hz, 2H), 2.77–2.67 (m, 1H), 1.14 (s, *J* = 6.9 Hz, 6H); ^13^C NMR (125 MHz, DMSO-*d_6_*) δ 159.9, 150.4, 147.1, 144.0, 136.5, 131.1, 127.0, 123.8, 122.7, 121.7, 119.9, 118.3, 115.0, 112.8, 111.6, 38.7, 32.9, 24.7; HRMS (ESI): *m/z* calcd for [M+H]^+^ C_21_H_20_N_4_O_2_ 361.1665; found 361.1681.

**6-(4-((mesitylamino)methyl)-1H-1,2,3-triazol-1-yl)-2H-chromen-2-one**(**6g**) Yield: 82%; Yellow solid; m.p: 157–159 °C; ^1^H NMR (500 MHz, DMSO-*d_6_*) δ 8.67 (s, 1H), 8.32 (s, 1H), 8.16 (d, *J* = 9.5 Hz, 1H), 8.12–8.08 (m, 1H), 7.63 (d, *J* = 8.8 Hz, 1H), 6.75 (s, 2H), 6.64 (d, *J* = 9.6 Hz, 1H), 4.19 (s, 2H), 2.22 (s, 6H), 2.15 (s, 3H); ^13^C NMR (125 MHz, DMSO-*d_6_*) δ 159.9, 153.3, 148.1, 144.0, 143.2, 133.3, 130.7, 130.3, 129.4, 123.7, 121.5, 120.0, 119.8, 118.3, 118.1, 43.3, 20.7, 18.6; HRMS (ESI): *m/z* calcd for [M+H]^+^ C_21_H_20_N_4_O_2_ 361.1665; found 361.1681.

**6-(4-(((4-phenoxyphenyl)amino)methyl)-1H-1,2,3-triazol-1-yl)-2H-chromen-2-one**(**6h**) Yield: 71%; Yellow solid; m.p: 172–174 °C; ^1^H NMR (500 MHz, DMSO-*d_6_*) δ 8.75 (s, 1H), 8.34 (d, *J* = 2.6 Hz, 1H), 8.16 (d, *J* = 9.6 Hz, 1H), 8.13 (dd, *J* = 8.9, 2.6 Hz, 1H), 7.63 (d, *J* = 8.9 Hz, 1H), 7.32–7.27 (m, 2H), 7.00 (t, *J* = 7.3 Hz, 1H), 6.86 (d, *J* = 8.7 Hz, 3H), 6.73 (d, *J* = 8.9 Hz, 2H), 6.64 (d, *J* = 9.6 Hz, 1H), 6.17 (t, *J* = 5.7 Hz, 1H), 4.40 (d, *J* = 5.7 Hz, 2H); ^13^C NMR (125 MHz, DMSO-*d_6_*) δ 159.9, 159.2, 153.3, 147.6, 146.4, 145.7, 144.0, 133.3, 130.1, 123.8, 122.3, 121.7, 121.4, 120.0, 119.9, 118.3, 118.1, 117.0, 113.8; HRMS (ESI): *m/z* calcd for [M+H]^+^ C_24_H_18_N_4_O_3_ 411.1457; found 411.1470.

**6-(4-(((3-nitrophenyl)amino)methyl)-1H-1,2,3-triazol-1-yl)-2H-chromen-2-one**(**6i**) Yield: 72%; Orange solid; m.p: 230–234 °C; ^1^H NMR (500 MHz, DMSO-*d*_6_) δ 8.77 (s, 1H), 8.33 (d, *J* = 2.6 Hz, 1H), 8.15 (d, *J* = 9.6 Hz, 1H), 8.11 (dd, *J* = 8.9, 2.6 Hz, 1H), 7.63 (d, *J* = 8.9 Hz, 1H), 7.49 (t, *J* = 2.2 Hz, 1H), 7.42–7.33 (m, 2H), 7.11 (d, *J* = 7.8 Hz, 1H), 6.99 (t, *J* = 5.7 Hz, 1H), 6.64 (d, *J* = 9.6 Hz, 1H), 4.51 (d, *J* = 5.7 Hz, 2H); ^13^C NMR (125 MHz, DMSO-*d*_6_) δ 159.9, 153.3, 149.8, 149.2, 146.7, 144.0, 133.3, 130.5, 123.9, 121.8, 120.0, 118.9, 118.3, 118.1, 110.8, 106.2, 38.7; HRMS (ESI): *m/z* calcd for [M+H]^+^ C_18_H_14_N_5_O_4_ 364.1046; found 364.1045.

**6-(4-(((3-bromophenyl)amino)methyl)-1H-1,2,3-triazol-1-yl)-2H-chromen-2-one**(**6j**) Yield: 72%; Yellow solid; m.p: 133–136 °C; ^1^H NMR (500 MHz, DMSO-*d_6_*) δ 8.73 (s, 1H), 8.33 (d, *J* = 2.6 Hz, 1H), 8.15 (d, *J* = 9.6 Hz, 1H), 8.12 (dd, *J* = 8.9, 2.6 Hz, 1H), 7.63 (d, *J* = 8.9 Hz, 1H), 7.03 (t, *J* = 8.0 Hz, 1H), 6.86 (t, *J* = 1.9 Hz, 1H), 6.70 (dd, *J* = 7.9, 1.0 Hz, 1H), 6.67 (dd, *J* = 8.2, 1.8 Hz, 1H), 6.64 (d, *J* = 9.6 Hz, 1H), 4.41 (d, *J* = 5.7 Hz, 2H); ^13^C NMR (125 MHz, DMSO-*d_6_*) δ 159.9, 153.3, 150.4, 147.1, 144.0, 133.3, 131.2, 123.8, 122.7, 121.7, 120.0, 119.9, 118.8, 118.3, 118.1, 114.9, 111.6, 38.7; HRMS (ESI): *m/z* calcd for [M+H]^+^ C_18_H_13_BrN_4_O_2_ 397.0300; found 399.0284.

**6-(4-(((3-trifluoromethyl)phenyl)amino)methyl)-1H-1,2,3-triazol-1-yl)-2H-chromen-2-one**(**6k**) Yield: 76%; Yellow solid; m.p: 246–248 °C; ^1^H NMR (500 MHz, DMSO-*d_6_*) δ 8.76 (s, 1H), 8.33 (d, *J* = 2.4 Hz, 1H), 8.15 (d, *J* = 9.6 Hz, 1H), 8.11 (dd, *J* = 8.9, 2.5 Hz, 1H), 7.63 (d, *J* = 8.9 Hz, 1H), 7.30 (t, *J* = 7.9 Hz, 1H), 6.99–6.92 (m, 2H), 6.85 (d, *J* = 7.6 Hz, 1H), 6.72 (t, *J* = 5.6 Hz, 1H), 6.64 (d, *J* = 9.6 Hz, 1H), 4.47 (d, *J* = 5.7 Hz, 2H); ^13^C NMR (125 MHz, DMSO-*d_6_*) δ 159.9, 153.3, 149.2, 147.0, 144.0, 133.3, 130.3, 126.0, 122.8 (d, *J* = 260.9 Hz), 120.0, 119.9, 118.3, 118.1, 115.9, 112.4, 108.8, 38.7; HRMS (ESI): *m/z* calcd for [M+H]^+^ C_19_H_13_F_3_N_4_O_2_ 387.1069; found 387.1194.

**6-(4-(((4-fluorophenyl)amino)methyl)-1H-1,2,3-triazol-1-yl)-2H-chromen-2-one**(**6l**) Yield: 73%; Yellow solid; m.p: 210–212 °C; ^1^H NMR (500 MHz, DMSO-*d_6_*) δ 8.71 (s, 1H), 8.32 (d, *J* = 2.6 Hz, 1H), 8.15 (d, *J* = 9.5 Hz, 1H), 8.11 (dd, *J* = 8.9, 2.7 Hz, 1H), 7.62 (d, *J* = 8.9 Hz, 1H), 6.96–6.91 (m, 2H), 6.70–6.65 (m, 2H), 6.63 (d, *J* = 9.6 Hz, 1H), 6.08 (t, *J* = 5.8 Hz, 1H), 4.37 (d, *J* = 5.8 Hz, 2H); ^13^C NMR (125 MHz, DMSO-*d_6_*) δ 159.9, 155.0 (d, *^1^J* = 231.2 Hz), 153.3, 147.5, 145.5, 144.0, 133.3, 123.8, 121.7, 120.0, 119.9, 118.2 (d, *^2^J* = 23.1 Hz), 115.7 (d, *^3^J* = 22.0 Hz), 113.6, 113.5; HRMS (ESI): *m/z* calcd for [M+H]^+^ C_18_H_13_FN_4_O_2_ 337.1101; found 337.1097.

**6-(4-((m-tolylamino)methyl)-1H-1,2,3-triazol-1-yl)-2H-chromen-2-one**(**6m**) Yield: 75%; Yellow solid; m.p: 220–223 °C; ^1^H NMR (500 MHz, DMSO-*d_6_*) δ 8.69 (s,1H), δ 8.32 (d, *J* = 2.5 Hz, 1H), 8.15 (d, *J* = 9.6 Hz, 1H), 8.11 (dd, *J* = 8.9, 2.6 Hz, 1H), 7.62 (d, *J* = 8.9 Hz, 1H), 6.97 (t, *J* = 7.7 Hz, 1H), 6.63 (d, *J* = 9.6 Hz, 1H), 6.53–6.46 (m, 2H), 6.39 (d, *J* = 7.3 Hz, 1H), 6.03 (t, *J* = 5.8 Hz, 1H), 4.39 (d, *J* = 5.8 Hz, 2H), δ 2.19 (s, 3H); ^13^C NMR (125 MHz, DMSO-*d_6_*) δ 159.9, 153.3, 148.8, 147.7, 144.0, 138.3, 133.4, 129.2, 123.8, 121.6, 120.0, 119.9, 118.3, 118.1, 117.6, 113.5, 110.0, 39.0, 21.8; HRMS (ESI): *m/z* calcd for [M+H]^+^ C_19_H_17_N_4_O_2_ 333.1352; found 333.1306.

**6-(4-(((3,5-dimethylphenyl)amino)methyl)-1H-1,2,3-triazol-1-yl)-2H-chromen-2-one**(**6n**) Yield: 71%; Yellow solid; m.p: 191–194 °C; ^1^H NMR (500 MHz, DMSO-*d_6_*) δ 8.32 (d, *J* = 2.5 Hz, 1H), 8.15 (d, *J* = 9.6 Hz, 1H), 8.11 (dd, *J* = 8.9, 2.6 Hz, 1H), 7.62 (d, *J* = 8.9 Hz, 1H), 6.63 (d, *J* = 9.6 Hz, 1H), 5.95 (t, *J* = 5.8 Hz, 1H), 4.37 (d, *J* = 5.8 Hz, 2H); ^13^C NMR (125 MHz, DMSO-*d_6_*) δ 160.0, 153.3, 148.7, 147.8, 144.0, 138.1, 133.3, 123.8, 121.5, 120.0, 119.9, 118.7, 118.3, 118.1, 110.8, 39.0, 21.7; HRMS (ESI): *m/z* calcd for [M+H]^+^ C_20_H_19_N_4_O_2_ 347.1508; found 347.1493.

**6-(4-(((3-chlorophenyl)amino)methyl)-1H-1,2,3-triazol-1-yl)-2H-chromen-2-one**(**6o**) Yield: 74%; Yellow solid; m.p: 134–137 °C; ^1^H NMR (500 MHz, DMSO-*d_6_*) δ 8.74 (s, 1H), 8.33 (d, *J* = 2.6 Hz, 1H), 8.15 (d, *J* = 9.6 Hz, 1H), 8.11 (dd, *J* = 8.9, 2.6 Hz, 1H), 7.62 (d, *J* = 8.9 Hz, 1H), 7.09 (t, *J* = 8.0 Hz, 1H), 6.71 (t, *J* = 2.1 Hz, 1H), 6.65–6.62 (m, 2H), 6.58–6.53 (m, 2H), 4.41 (d, *J* = 5.7 Hz, 2H); ^13^C NMR (125 MHz, DMSO-*d_6_*) δ 159.9, 153.3, 150.2, 147.1, 144.0, 134.0, 133.3, 130.8, 123.8, 121.7, 120.0, 119.9, 118.3, 118.1, 115.9, 112.0, 111.4, 38.7; HRMS (ESI): *m/z* calcd for [M+H]^+^ C_18_H_13_ClN_4_O_2_ 353.0805; found 353.0763.

**6-(4-(((2-iodophenyl)amino)methyl)-1H-1,2,3-triazol-1-yl)-2H-chromen-2-one**(**6p**) Yield: 67%; Yellow solid; m.p: 165–167 °C; ^1^H NMR (500 MHz, DMSO-*d_6_*) δ 8.69 (s, 1H), 8.32 (d, *J* = 2.6 Hz, 1H), 8.16–8.10 (m, 2H), 7.65 (dd, *J* = 7.8, 1.5 Hz, 1H), 7.61 (d, *J* = 8.9 Hz, 1H), 7.21–7.16 (m, 1H), 6.73 (dd, *J* = 8.2, 1.2 Hz, 1H), 6.63 (d, *J* = 9.6 Hz, 1H), 6.42 (td, *J* = 7.6, 1.4 Hz, 1H), 5.47 (t, *J* = 5.8 Hz, 1H), 4.55 (d, *J* = 5.8 Hz, 2H); ^13^C NMR (125 MHz, DMSO-*d_6_*) δ 159.9, 153.3, 147.5, 147.1, 144.0, 139.3, 133.3, 129.7, 124.0, 121.6, 120.0, 119.9, 119.0, 118.2, 118.1, 111.4, 85.4; HRMS (ESI): *m/z* calcd for [M+H]^+^ C_18_H_13_IN_4_O_2_ 445.0161; found 445.0149.

**6-(4-(((4-chloro-2-nitrophenyl)amino)methyl)-1H-1,2,3-triazol-1-yl)-2H-chromen-2-one** (**6q**) Yield: 74%; Yellow solid; m.p: 213–215 °C; ^1^H NMR (500 MHz, DMSO-*d_6_*) δ 8.74 (s, 2H), δ 8.31 (d, *J* = 2.5 Hz, 1H), 8.14 (d, *J* = 9.6 Hz, 1H), 8.12–8.08 (m, 2H), 7.65–7.55 (m, 2H), 7.19 (d, *J* = 9.3 Hz, 1H), 6.63 (d, *J* = 9.6 Hz, 1H), 4.82 (d, *J* = 6.0 Hz, 2H); ^13^C NMR (125 MHz, DMSO-*d_6_*) δ 159.9, 153.4, 146.0, 144.0, 136.7, 133.2, 131.9, 125.4, 124.0, 121.8, 120.0, 119.9, 119.3, 118.3, 118.1, 117.4, 38.5; HRMS (ESI): *m/z* calcd for [M+H]^+^ C_18_H_13_ClN_5_O_4_398.0656; found 398.0631.

**6-(4-(((2,3-dimethylphenyl)amino)methyl)-1H-1,2,3-triazol-1-yl)-2H-chromen-2-one**(**6r**)Yield: 78%; Yellow solid; m.p: 220–222 °C; ^1^H NMR (500 MHz, DMSO-*d_6_*) δ 8.65 (s, 1H), δ 8.31 (d, *J* = 2.6 Hz, 1H), 8.15–8.09 (m, 2H), 7.60 (d, *J* = 9.0 Hz, 1H), 6.87 (t, *J* = 7.8 Hz, 1H), 6.62 (d, *J* = 9.6 Hz, 1H), 6.48 (dd, *J* = 20.5, 7.7 Hz, 2H), 5.45 (t, *J* = 5.8 Hz, 1H), 4.46 (d, *J* = 5.7 Hz, 2H), δ 2.19 (s, 3H), δ 2.05 (s, 3H); ^13^C NMR (125 MHz, DMSO-*d_6_*) δ 159.9, 153.2, 148.2, 146.3, 144.0, 136.1, 133.3, 126.2, 123.8, 121.5, 120.8, 119.9, 119.8, 119.0, 118.2, 118.1, 108.3, 20.8, 13.1; HRMS (ESI): *m/z* calcd for [M+H]^+^ C_20_H_19_N_4_O_2_ 347.1508; found 347.1497.

**6-(4-(((3,5-difluorophenyl)amino)methyl)-1H-1,2,3-triazol-1-yl)-2H-chromen-2-one**(**6s**)Yield: 74%; Yellow solid; m.p: 204–206 °C; ^1^H NMR (500 MHz, DMSO-*d_6_*) δ 8.76 (s, 1H), δ 8.33 (d, *J* = 2.6 Hz, 1H), 8.16 (d, *J* = 9.6 Hz, 1H), 8.12 (dd, *J* = 8.9, 2.6 Hz, 1H), 7.63 (d, *J* = 8.9 Hz, 1H), 6.87 (t, *J* = 5.6 Hz, 1H), 6.64 (d, *J* = 9.6 Hz, 1H), 6.34 (dd, *J* = 10.8, 2.2 Hz, 2H), 6.26 (tt, *J* = 9.5, 2.2 Hz, 1H), 4.42 (d, *J* = 5.7 Hz, 2H); ^13^C NMR (125 MHz, DMSO-*d_6_*) δ 163.9 (d, *J* = 224.2 Hz), 159.9, 153.3, 151.5, 146.6, 144.0, 133.3, 123.9, 121.9, 120.0, 118.3, 118.1, 95.2, 91.0, 38.6; HRMS (ESI): *m/z* calcd for [M+H]^+^ C_20_H_19_N_4_O_2_ 355.1007; found 355.0988.

**6-(4-(((2-fluorophenyl)amino)methyl)-1H-1,2,3-triazol-1-yl)-2H-chromen-2-one**(**6t**) Yield: 71%; Yellow solid; m.p: 230–232 °C; ^1^H NMR (500 MHz, DMSO-*d_6_*) δ 8.70 (s, 1H), 8.32 (d, *J* = 2.6 Hz, 1H), 8.16–8.10 (m, 2H), 7.61 (d, *J* = 8.9 Hz, 1H), 7.03 (ddd, *J* = 12.3, 8.0, 1.4 Hz, 1H), 6.98–6.93 (m, 1H), 6.84–6.80 (m, 1H), 6.63 (d, *J* = 9.6 Hz, 1H), 6.57 (tdd, *J* = 7.8, 4.8, 1.6 Hz, 1H), 6.07–6.03 (m, 1H), 4.49 (d, *J* = 6.0 Hz, 2H); ^13^C NMR (125 MHz, DMSO-*d_6_*) δ 160.0, 153.3, 151.4 (d, *J* = 237.7 Hz), 147.5, 144.0, 136.7, 133.3, 125.2, 123.9, 121.6, 119.9, 118.3, 118.1, 116.4, 114.7, 112.7, 38.7; HRMS (ESI): *m/z* calculated for [M+H]^+^ C_18_H_13_FN_4_O_2_ 337.3278; found 337.1092.

### 3.2. CA Inhibition

An SX.18V-R Applied Photophysics (Oxford, UK) stopped flow instrument has been used to assay the CA catalyzed CO_2_ hydration activity [26]. Phenol Red (at a concentration of 0.2 mM) has been used as an indicator, working at an absorbance maximum of 557 nm, with 10 mM Hepes (pH 7.4) as a buffer, 0.1 M Na_2_SO_4_ or NaClO_4_ (for maintaining constant the ionic strength; these anions are not inhibitory in the used concentration), following the CA-catalyzed CO_2_ hydration reaction for a period of 5–10 s. Saturated CO_2_ solutions in water at 25 °C were used as substrate. Stock solutions of inhibitors were prepared at a concentration of 10 mM (in DMSO-water 1:1, *v/v*) and dilutions up to 0.01 nM done with the assay buffer mentioned above. At least 7 different inhibitor concentrations have been used for measuring the inhibition constant. Inhibitor and enzyme solutions were pre-incubated together for 10 min at room temperature prior to assay, in order to allow for the formation of the E-I complex. Triplicate experiments were done for each inhibitor concentration, and the values reported throughout the paper are the mean of such results. The inhibition constants were obtained by non-linear least squares methods using the *Cheng-Prusoff* equation, as reported earlier, and represent the mean from at least three different determinations. All CA isozymes used here were recombinant proteins obtained as reported earlier by our group and their concentration was of 2.5–12 nM in the assay system [27,28,29,30].

## 4. Conclusions

**Figure 2:**
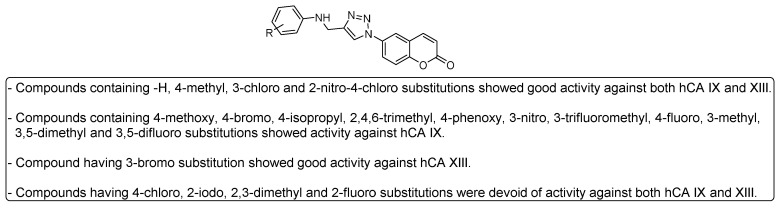
SAR of coumarin-linked 4-anilinomethyl-1,2,3-triazoles.

In the present study, two potent CA pharmacophores, coumarin and substituted 4-anilinomethyl-1,2,3-triazoles, were combined using a molecular hybridization approach and a series of new 6-substituted-coumarin derivatives **6a**–**t** was synthesized. The 1,2,3-triazole moiety was tethered to the C-6 position of the coumarin moiety. All the synthesized compounds were screened against four CA isoforms, I, II, IX and XIII. None of the compounds showed significant inhibition of the off-target CA isoforms, I and II. Most compounds showed good inhibition of CA IX (K_i_ < 100 nM), with compound **6e** showing the most effective inhibition (K_i_ = 36.3 nM). Against CA XIII, compounds **6a**, **6b**, **6j**, **6o** and **6q** exhibited good inhibitory profiles (K_i_ < 100 nM). Thus, these molecules can be further explored as a starting point for the design of potent and effective inhibitors against CA IX and CA XIII isoforms. The structure activity relationship SAR of the synthesized compounds has been depicted in Figure 2.

## Figures and Tables

**Table 1:** Inhibition of hCA isoforms I, II, IX and XIII with compounds **6a**–**t** and acetazolamide (AAZ) as standard inhibitor.

	K_i_ (nM) *				
Compound	Structure	hCA I	hCAII	hCA IX	hCA XIII
**6a**	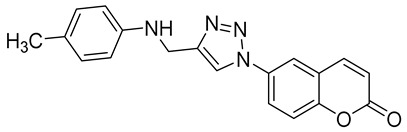	>50,000	>50,000	**48.4**	**92.6**
**6b**	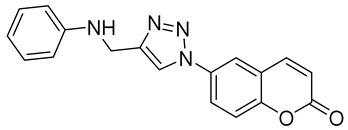	>50,000	>50,000	60.8	**90.1**
**6c**	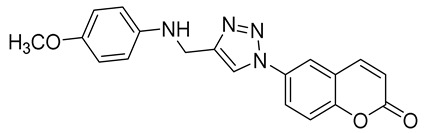	>50,000	>50,000	73.3	950.5
**6d**	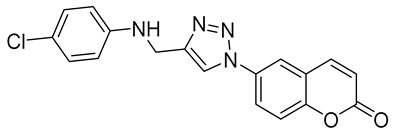	>50,000	>50,000	151.9	897.6
**6e**	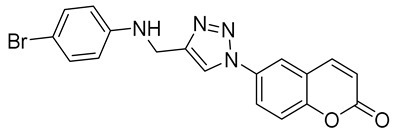	>50,000	>50,000	**36.3**	883.2
**6f**	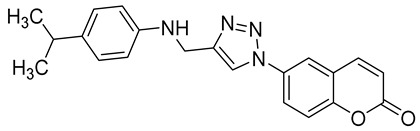	>50,000	>50,000	**45.0**	8149
**6g**	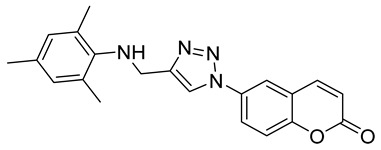	>50,000	>50,000	64.8	902.7
**6h**	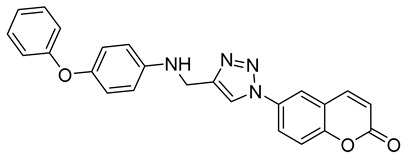	>50,000	>50,000	73.0	905.4
**6i**	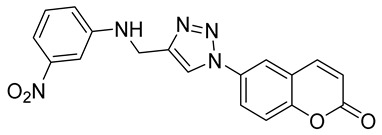	>50,000	>50,000	85.7	896.1
**6j**	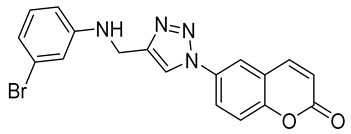	>50,000	>50,000	304.9	**91.8**
**6k**	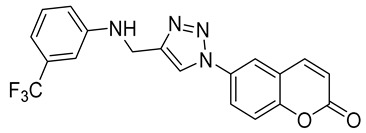	>50,000	>50,000	69.1	6426
**6l**	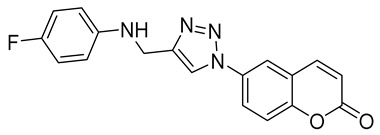	>50,000	>50,000	86.1	839.8
**6m**	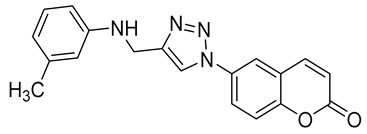	>50,000	>50,000	80.1	795.2
**6n**	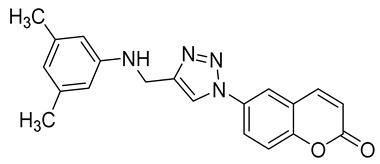	>50,000	>50,000	89.3	703.4
**6o**	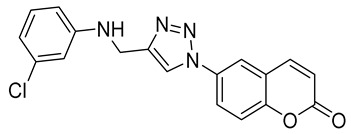	>50,000	>50,000	92.7	**95.4**
**6p**	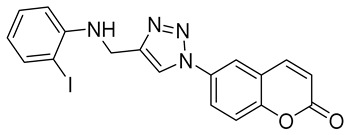	>50,000	>50,000	573.9	594.4
**6q**	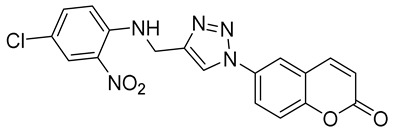	>50,000	>50,000	96.4	**96.7**
**6r**	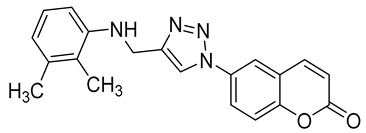	>50,000	>50,000	462.1	550.1
**6s**	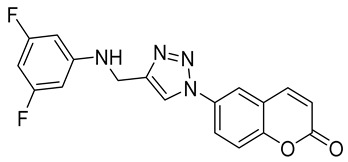	>50,000	>50,000	90.9	664.4
**6t**	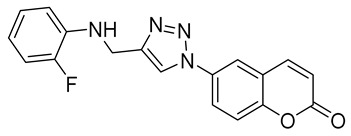	>50,000	>50,000	642.8	6034
**AAZ**		250	12.1	25.8	17.0

* Mean from 3 different assays, by a stopped flow technique (errors were in the range of ±5–10% of the reported values). The values in bold shows the active compounds.

## Data Availability

Not applicable.
